# P12 Unjustified use of antibiotics in wartime conditions in Ukraine

**DOI:** 10.1093/jacamr/dlad143.016

**Published:** 2024-01-03

**Authors:** Kiarina Myroniuk-Konstantynovych, Iryna Bondaruk, Tetiana Bevz, Yaroslav Demchyshyn, Tetyana Konstantynovych

**Affiliations:** National Pirogov Memorial Medical University, Vinnytsya, Ukraine; National Pirogov Memorial Medical University, Vinnytsya, Ukraine; National Pirogov Memorial Medical University, Vinnytsya, Ukraine; National Pirogov Memorial Medical University, Vinnytsya, Ukraine; National Pirogov Memorial Medical University, Vinnytsya, Ukraine

## Abstract

**Background:**

The Ministry of Health of Ukraine reported that every second patient who takes antibiotics does not need such treatment, and every third uses them for viral diseases. In Ukraine, during the war, there has formed a resonance between the availability of broad-spectrum antibiotics and their belonging to the non-recommended category according to the AWaRe classification 2023. Therefore, the fight against antibiotic resistance continues to be a leading task of modern medicine.

**Objectives:**

To investigate the expediency of use of ceftriaxone/levofloxacin among Ukrainian and foreigners settled in Ukraine.

**Subjects and methods:**

A total of 180 (55% Ukrainians, 45% foreigners) responders were examined by questionnaire for ceftriaxone/levofloxacin use over 1 year with specification of reasons, expediency of use, possible reason for cancellation/replacement of antibiotic therapy and preventable usage cases.

**Results:**

The sample included 63.9% women, 36.1% men, average age 18–25 years. The incidence of ceftriaxone/levofloxacin use was 58.97% among foreigners versus 12.74% among Ukrainians (*P*<0.001). The antibiotic use by physician’s prescription occurred among 28.3% foreigners and 92.3% Ukrainians (*P* < 0.05). The frequency of antibiotic use due to microbiological confirmation was significantly lower among foreign responders: 13.05% versus 76.9% in Ukrainians (*P* < 0.01). The reasons for choice of ceftriaxone/levofloxacin in the Ukrainian sample were acute respiratory infections 46%, fever 7.8% and diseases of bacterial origin 84.6%. At the same time foreigners used antibiotics for acute respiratory infections 41.3%, fever 19.7% and diseases of bacterial origin 63.1%. The analysis found that antibiotic use for ‘prophylaxis’ (mainly for viral diseases) was 2.95% among Ukrainians and 32.05% among foreigners (*P*<0.05). The predominant reasons for cancellation/replacement of antibiotic therapy, such as allergic reactions and antibiotic-associated diarrhoea, were found in 67.4% of foreigners and 7.69% of Ukrainians (*P*<0.05).

**Conclusions:**

The lack of antibiotic prescription on the basis of microbiological confirmation, self-limited choice of antibiotics and unreasonable use for diseases of non-bacterial origin can be factors that promote multiple resistance in future and complicate the natural clinical course of most common infectious diseases.

